# Quinine

**Published:** 1881-05

**Authors:** 


QUININE.—Extensively and, we might add, universally as quinine is prescribed, we feel sure the utmost boundaries of its usefulness have not yet been reached. Though entering into the prescriptions of physicians almost daily in every portion of the civilized world, there are some grave errors and misapprehensions entertained by not a few in regard to its physiological action upon the system in health and in disease, and who even attribute toxic properties to it in certain cases which it in no wise possesses, or is capable of producing. For example, it is thought, unwise and improper by some to prescribe it to a pregnant woman, lest it should produce uterine contractions and the final loss of the fœtus. Others, relying upon a misapprehension of its antipyretic effects, prescribe it in the pyrexia of fever. Both those ideas of its action are erroneous, as we know from long experience in the malarial regions of the Southern States. We have prescribed it for malaria and other troubles, to hundreds of pregnant females—white, yellow and black—for many years, and in every stage of fœtal development, from the time of conception to the end of utero-gestation, without ever observing toxic or unpleasant effects from its use in a single instance; and we need hardly add, that the doses necessary to combat Southern malarial fevers are sufficiently large to have shown toxic effects had any such belonged to it. It produces so little, if any, antipyretic effect upon the hot stage of fever, that it may be advantageously substituted by any one of a dozen better and more efficient articles in that stage. In fact, we would regard its use now in the hot stage of fever as an useless expenditure both of time and money. We have prescribed it in the hot stage of yellow, typhoid, remitting and intermitting fever, in full doses and repeatedly, without observing any curtailment of the paroxysm, and with the only obviously marked effect of and increase of cerebral pain and uneasiness. The foregoing misapprehensions of the action of quinia upon the system have been brought prominently forward, as they are more widespread than others which might be adduced. It will neither relieve the pyrexia of fever nor induce a chill; the theory of our homœopathic confreres to the contrary notwithstanding.

It is an *anti-periodic* in the highest and most emphatic sense of that word, and as such is adapted to the scientific treatment of a larger number of diseases than any other single article of the materia medica. We do not publish the letter asking for our experience with quinine, for want of space; and have been compelled to make our remarks as brief as possible for the same reason. We will endeavor to return to the subject in a future issue of the journal.

